# A Novel Homozygous Mutation of the *AIRE* Gene in an APECED Patient From Pakistan: Case Report and Review of the Literature

**DOI:** 10.3389/fimmu.2018.01835

**Published:** 2018-08-13

**Authors:** Marsha Pellegrino, Emanuele Bellacchio, Rudina Dhamo, Federica Frasca, Corrado Betterle, Alessandra Fierabracci

**Affiliations:** ^1^Infectivology and Clinical Trials Research Division, Bambino Gesù Children’s Hospital, Rome, Italy; ^2^Molecular Genetics and Functional Genomics, Genetics and Rare Diseases Research Division, Bambino Gesù Children’s Hospital, Rome, Italy; ^3^Pediatric Division, Jesi Hospital, Jesi, Italy; ^4^Endocrine Unit, Department of Medicine (DIMED), University of Padua, Padua, Italy

**Keywords:** APECED, *AIRE* gene, genotype/phenotype correlation, mutation functional analysis, epidemiology

## Abstract

Autoimmune-poly-endocrinopathy-candidiasis–ectodermal-dystrophy syndrome (APECED) is a rare monogenic recessive disorder caused by mutations in the autoimmune regulator (*AIRE*) gene. Criteria for the diagnosis of APECED are the presence of two of the following disorders: chronic mucocutaneous candidiasis (CMC), chronic hypoparathyroidism (CHP), and Addison’s disease. APECED develops at high incidence in Finns, Sardinians, and Iranian Jews and presents with a wide range of clinical phenotypes and genotypes. In this manuscript, we report the clinical, endocrinological, and molecular features of a 16-year-old female patient from Pakistan living in Italy and presenting the major APECED clinical manifestations CMC, CHP, and primary adrenal insufficiency. Premature ovarian failure, chronic bronchopneumopathy, vitiligo, Hashimoto’s thyroiditis emerged as associated diseases. In our patient, *AIRE* gene screening revealed the novel c.396G>C (p.Arg132Ser; p.R132S) mutation in homozygosity thus confirming APECED diagnosis. This is the first reported mutation within the nuclear localization signal (NLS) that is associated with APECED. The NLS mutation affects the nuclear import of classical transcription factors through nuclear pore by recognition of nuclear import receptors, the importin α molecules. By displaying crystal structures of the peptide containing the KRK basic residue cluster bound to α importins, we show that p.R132S replacement in 131-KRK-133 does not reproduce these interactions. Thus, we propose that the novel mutation exerts its pathogenetic effect by impairing the nuclear import of the Aire protein. The present case report is added to a limited series of Pakistani APECED patients who we reviewed from the scientific literature, mostly diagnosed on clinical findings.

## Introduction

**Figure 1:**
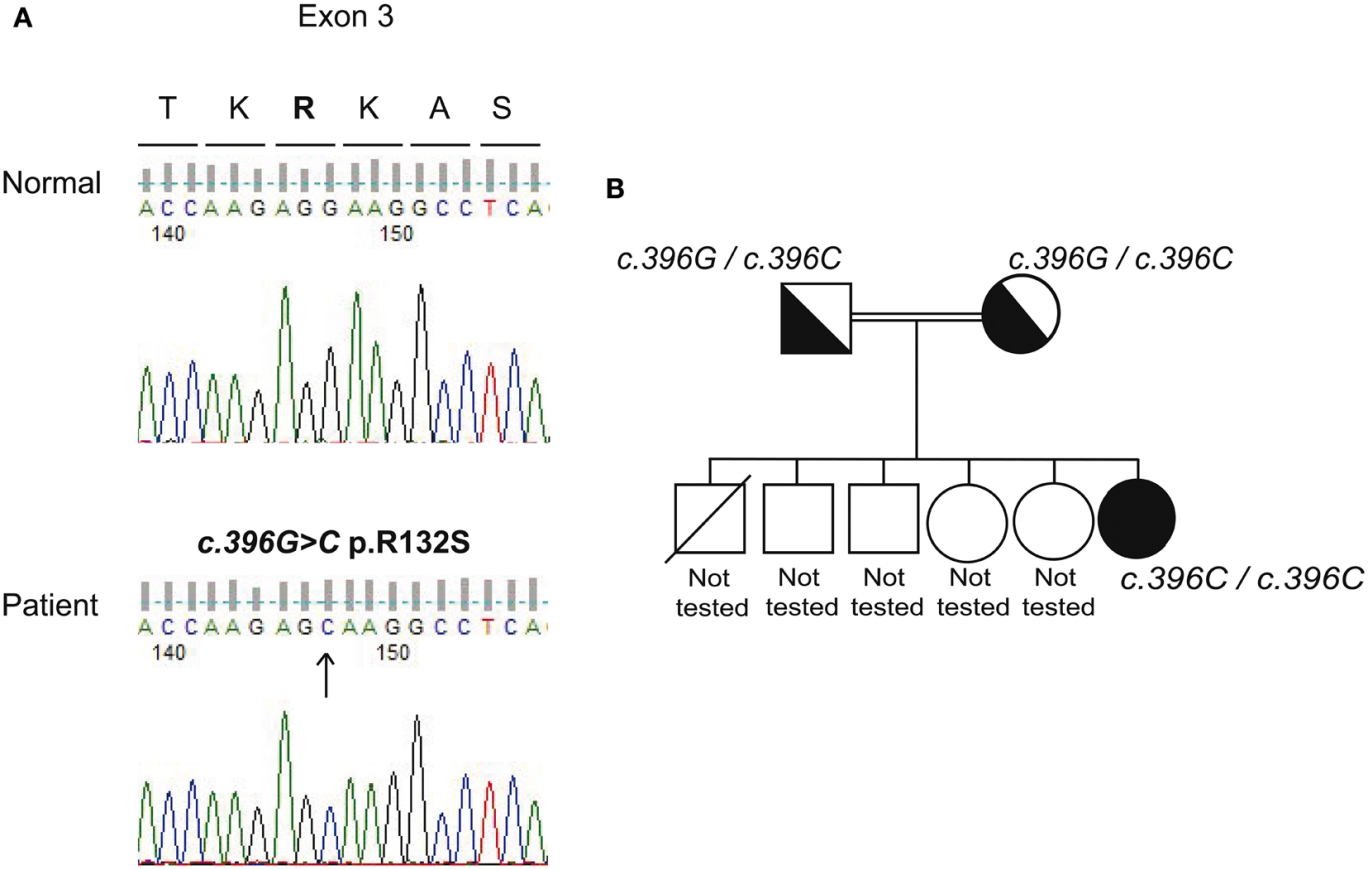
Genetic analysis of the *AIRE* gene. **(A)** Electropherograms of representative fragments of exon 3 of the *AIRE* gene relative to a normal control and the patient. The analysis was performed after informed consent. The wild-type allele for exon 3 is shown for the control (upper panel). The patient (bottom panel) is homozygous revealing two copies of the mutated c.396G>C allele (exon 3). Fifty normal controls tested negative for the novel mutation. **(B)** The phylogenetic tree relative to the family studies showing the heterozygous state for the novel c.396G>C mutation in both parents and the homozygosity in the patient’s DNA.

A 16-year-old female patient (weight at birth 3 kg) was the third child of consanguineous parents, without any family history for autoimmune diseases. Older brother died for unknown reason at the age of 11 years, the four living children, two males and two females, are in apparently good health at the age of 19, 6, 11.5, and 10, respectively (Figure 1). The patient and her family come originally from Pakistan. From the age of 3 years, the patient and her family live in continental Italy.

From the age of 2 years, the patient was affected by onichodystrophy at the right thumb. The patient was diagnosed for chronic hypoparathyroidism (CHP) at the age of 8 years and started therapy with calcium carbonate and calcitriol *per os*; she was diagnosed for Addison’s disease (AD) from the age of 14.7 years and started therapy with hydrocortisone and fludrocortisone *per os*. Currently, the patient presents dehydration episodes with hyponatremia. The patient is also affected by chronic obstructive bronchopneumopathy and dust mites allergy, in therapy with fluticasone and salbutamol intranasal administration, vitiligo, and Hashimoto’s thyroiditis with normal thyroid function. Menarche appeared at 13.2 years of age with a hypergonadotropic hypogonadism at the age of 14.8. At present, the patient is under progesterone treatment. At the age of 14.7 years, oral and fecal swab and scraping sample cultures obtained from the patient’s nails showed *Candida albicans* proliferation suggesting the diagnosis of chronic mucocutaneous candidiasis (CMC) at present under miconazole treatment. Organ and non-organ specific autoantibodies (Abs) screening revealed positivity for thyroid peroxidase (TPO) and thyroglobulin (Tg) Abs indicative of Hashimoto’s thyroiditis, for 21 hydroxylase (21OH Abs) revealing autoimmune AD, for 17αOH, cytochrome P450 Side Chain Cleavage Enzyme (P450scc Abs) indicative of a premature ovarian failure due to lymphocytic oophoritis, for aromatic l-amino acid decarboxylase (AADC) Abs indicative of autoimmune enteropathy (1, 2), although the patient does not present gastrointestinal dysfunction (ID), and for type 1 interferon (IFN) Abs, i.e., IFN-ω (test carried out at FIRS Laboratories, Cardiff, UK) (3) and IFN-α (test carried out at Laboratory of Human Genetics of Infectious Diseases, INSERM, Paris, France) (Figure S1 in Supplementary Material, Table 1) (4) which are typical of APECED patients. CMC-related neutralizing IL-17A, IL-17F, and IL-22 Abs tested negative (4, 5). Abs to tryptophan hydroxylase (TPH) (1), to acetylcholine receptor, to nuclear (ANA), to mitochondrial (AMA), to smooth muscle (SMA), to liver-kidney microsomal, and to parietal cell (PCA) were tested and showed to be negative (Table 1). Interestingly, the serum of both parents was tested and proved to be positive for IFN-ω Abs at low titer (7.0 IU cutoff ≤5 IU) in absence of autoimmune disease manifestations.

**Table 1:** Data related to the present case report and published Pakistani APECED patients.

Age	Clinical manifestation/therapy	Abs profile	Laboratory and instrumental parameters
From 2 years old	Onychomycosis at right thumb		Mycelial fungi cultures negative

8 years old	Hypoparathyroidism under calcium carbonate and calcitriol treatment		**PTH ↓, Ca ↓, P↑**

10.5 years old	Chronic allergic obstructive bronchopneumopathy under treatment with fluticasone and salbutamol		**Dust mites positive prick test**

14 years old	Vitiligo		

14.6 years old	Secondary amenorrhea under progesterone treatment		**FSH ↑, LH↑, progesterone↓, estradiol↓**

14.7 years	Primary adrenal insufficiency under hydrocortisone and fludrocortisone treatment		**ACTH ↑, basal cortisol ↓, cortisoluria in 24 h ↓, ACTH test: no cortisol increase, aldosterone↓, plasma renin activity ↑, DHEAS↓, Na ↓, K ↑**

	Chronic mucocutaneous candidiasis under miconazole treatment		**Oral, fecal swab confirming ***Candida albicans*** proliferation**

	Hashimoto’s thyroiditis in euthyroidism	**TPO Abs 79 cutoff ≤25, Tg Abs 418 cutoff ≤40**, ANA, AMA, ASMA, LKMA neg, PCA neg	

	Dental enamel dysplasia	**21OH Abs 0.7 cutoff ≤0.4 IU**, **17αOH Abs 5.9 cutoff ≤1 IU, P450scc Abs >32 cutoff ≤1 IU, AADC Abs 102.4 cutoff ≤2.7 IU**, AChR Abs neg, TPH Abs neg, **IFN-ω Abs 76 cutoff ≤5 IU, IFN-α Abs pos**, IFN-17A Abs neg, IFN-17F Abs neg, IL-22 Abs neg	**Homozygous c.396G>C *AIRE* gene mutation**

*Clinical presentation, laboratory and instrumental parameters of the APS1 patient (altered parameters are in bold)*.

*Pos, positive; neg, negative; PTH, parathyroid hormone; Ca, serum calcium; P, serum phosphorus; FSH, follicle-stimulating hormone; LH, luteinizing hormone; ACTH, adrenocorticotropic hormone; DHEAS, dehydroepiandrosterone; Na, serum sodium; K, serum potassium; IL, interleukin; AChR, acetylcholine receptor; LKMA, liver-kidney microsomal; TPH, tryptophan hydroxylase; Abs, autoantibodies; TPO, thyroid peroxidase; IFN, interferon*.

### Identification of c.396G>C Mutation and Its Effect on Nuclear Localization Signal (NLS) Function

The novel autoimmune regulator *(AIRE)* gene c.396G>C (p.Arg132Ser) (exon 3) mutation in homozygosity was detected confirming APECED diagnosis (Figure 1). Homozygous c.1578T>C (p.Asp526=, rs1133779) (exon 14) synonymous variant was also detected.

**Figure 2:**
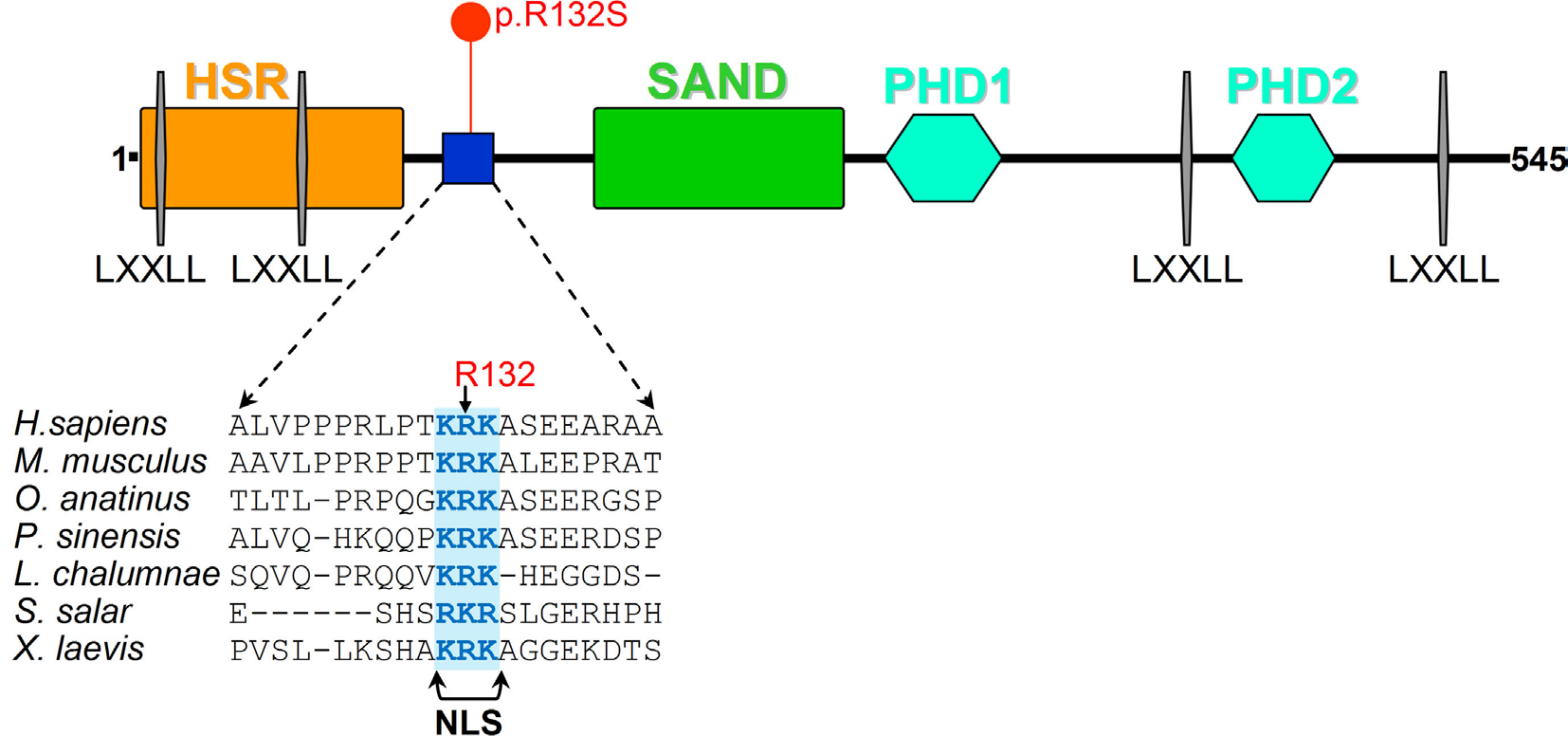
Aire protein analysis representation. Schematic representation of Aire protein is shown along with the protein sequence alignment of the region surrounding the site of the p.R132S amino acid replacement among vertebrates. The functional nuclear localization signal (NLS) (6) is highlighted.

Arg132 resides in the Aire protein region that comprises the functional NLS responsible for the protein correct subcellular localization and thus its activity (Figure 2). To show how the p.R132S replacement in 131-KRK-133 influences the function of the NLS, we have displayed crystal structures of an NLS peptide containing the identical KRK basic residue cluster bound to α-importins. As observed in Figure 2 and Figure S2 in Supplementary Material, the central arginine (corresponding to Aire Arg 132) makes a multiplicity of interactions with α-importins regardless the KRK cluster is bound to the minor or the major NLS-binding site. These interactions cannot be reproduced by replacement with the tiny and uncharged serine residue.

## Background

The autoimmune-poly-endocrinopathy-candidiasis–ectodermal-dystrophy/dysplasia (APECED) or autoimmune polyendocrine syndrome type 1 is a rare monogenic recessive autoimmune syndrome caused by mutations in the *AIRE* gene [reviewed in Ref. (7)]. This gene encodes for the thymus-enriched transcription factor Aire responsible for central immune tolerance being involved both in the deletion of autoreactive T cells by negative selection and in controlling processing/presentation of autoantigens within the thymus [reviewed in Ref. (7, 8)]. Aire protein is not only detected in immunologically important tissues as thymic medullary epithelial cells but also in peripheral blood and in the monocyte/dendritic cell lineage (9–12). This diverse expression could explain the variability of symptoms in APECED patients. Among the immunological abnormalities, a defect in CD4+CD25+ regulatory T cells, key mediators in peripheral tolerance and prevention of autoimmunity, contributes to APECED pathogenesis [reviewed in Ref. (7, 13–15)].

The old criteria for the clinical diagnosis of APECED were based on the presence of at least two of the following disorders: CMC, CHP, and primary adrenal insufficiency or AD. Furthermore, 78% of APECED patients present non-endocrine manifestations before the first endocrinopathy is manifested (16). New criteria for the diagnosis of APECED were introduced in 2009 (17) based on genetic and immunological features of the syndrome, independently from the phenotypical clinical manifestations.

APECED develops at high incidence (1:9,000–1:25,000) in Finns, Sardinians, and Iranian Jews. These ethnicities present the “signature” homozygous *AIRE* mutations c.769C>T (R257X), c.415C>T (R139X), and c.254A>G (Y85C), respectively (16, 18–20). More than 80–90% of APECED patients present CMC as first symptom (16, 19, 20). APECED is detected at lower incidences (approximately 1:90,000–3:100,000) and with greater genetic variability (17, 21–31) in many European countries such as Slovenia, Slovakia, Russia, Great Britain, France, Italy, Ireland, Poland, and other Scandinavian countries (i.e., Norway, Sweden). In American APECED patients was observed an enrichment of organ-specific non-endocrine manifestations (40–80% of patients) appearing early in life, compared with European APECED cohorts. The diverse clinical picture included urticarial eruption, autoimmune hepatitis, autoimmune gastritis, autoimmune ID, autoimmune pneumonitis, and SjÖgren-like syndrome (32).

In this manuscript, we report a female APECED patient from Pakistan with a newly discovered homozygous *AIRE* gene mutation and review the literature on published APECED Pakistani patients.

## Discussion

Among the classic clinical features, this Pakistani female patient presented all fundamental APECED symptoms of the classic triad: CMC, CHP, and AD. Interestingly, circulating 21OH, 17αOH, P450scc, AADC, TPO, Tg, and IFN-ω Abs were tested and found positive (Table 1). Interestingly, the serum of both parents also turned out positive for IFN-ω Abs at low titer without any autoimmune disorder being present. The presence of the novel c.396G>C (p.Arg132Ser) homozygous mutation of the *AIRE* gene confirms the diagnosis of APECED syndrome based on the new criteria (17, 32).

To the best of our knowledge, a limited series of APECED patients have been reported in the Pakistani population, mostly diagnosed on clinical findings. We reviewed the literature published from 2006 to date (Table 2) (5, 33–35) on a series of six APECED patients including the present case report (female/male ratio of 4:2, median age at referral 15 years; range 8–17 years) with early disease onset (median 8 years, age range 6–14 years, based on the appearance of the first component of the triad) and severe phenotype (on average, four manifestations per patient) (Table 2).

**Table 2:** Clinical and genetic features of a small series of Pakistani APECED patients reported between 2006 and 2018 including the present case report.

Patients	Sex	Age at referral	Age of first symptom	*AIRE* mutation	Major clinical manifestations related to APECED	Other clinical manifestations	Reference
1	F	15	8	*Exon 3*: homozygous c.396G>C p.Arg132Ser	Adrenal insufficiency, chronic hypoparathyroidism, chronic mucocutaneous candidiasis	Vitiligo, POF, Hashimoto’s thyroiditis, enamel dysplasia, chronic bronchopneumopathy	Present report
				*Exon 14*: c.1578T>C p.Asp526=			

2	F	17	Childhood (<14)	NA	Chronic mucocutaneous candidiasis (oral ulcers), Addison’s disease (hyperpigmentation)		(33)

3	F	8	6	Not tested	Chronic mucocutaneous candidiasis, Addison’s disease (hyperpigmentation), hypoparathyroidism	Nail dystrophy, vitiligo, epileptiform seizures, *Alcaligenes faecalis* septicemia	(34)

4	NA	NA	NA	*Exon 8*: homozygous c.967_979del13	NA	NA	(5)

5	NA	NA	NA	NA	NA	NA	(35)

6	F	NA	NA	NA	NA	Renal failure	Unpublished (Harachi, Pakistan) 2010

*NA, information not available; POF, premature ovarian failure*.

Interestingly, the novel diagnostic c.396G>C variant of the present case report is the first NLS localized mutation within the *AIRE* gene that is associated with APECED (6). In this regard, the Aire protein contains several functional domains, in addition to NLS, two plant homeodomain-type zinc fingers, four nuclear receptor-binding LXXLL motifs, homogeneously staining region (HSR), and SAND [Sp100, speckled protein 100 kDa, NucP41/75, nuclear phosphoprotein 41/75, DEAF-1 (deformed epidermal autoregulatory factor-1)] domains [reviewed in Ref. (6, 36)]. Aire acts as a transcriptional transactivator *in vitro* [reviewed in Ref. (6, 7, 37)] by DNA binding and interaction with the common coactivator CREB (cAMP-response element binding)-binding protein (CBP) [reviewed in Ref. (6, 38)].

Aire protein, both endogenous and transiently expressed, is predominantly localized in the cell nucleus where it regulates the expression of peripheral tissue antigens by different mechanisms either sequence-specific and/or epigenetic (9). When transiently expressed, Aire can be found in the cytoplasm attached to intermediate filaments or complexed within aggregates (9); therefore, in cells Aire can be shuttled between the nucleus and the cytoplasm. A nuclear export signal is also contained within the Aire HSR domain. The NLS is bipartite and constituted of amino acids 110–114 and 131–133 (6). Classic transcription factors containing typical NLSs are transported into the nucleus through nuclear pores by recognition of nuclear import receptors (39), the importin α molecules. The generated complex is bound by importin β that mediates its translocation into the nucleus in a Ran-GTPase-dependent manner (39). Incani et al. (40) proposed that lysine’s acetylation increased Aire stability inside the nucleus; conversely, Aire interaction with deacetylase complexes halts protein transactivation activity making it susceptible to proteosomal degradation.

In this work, we present the Aire p.R132S amino acid replacement that directly affects the central basic residue in the active NLS of Aire (amino acids 131–133) as demonstrated by Ilmarinen et al. (6). These authors identified the functional NLS region in Aire by carrying out mutagenesis analysis within the two adjacent clusters of basic amino acids 110-RKGRK-114 and 131-KRK-133 (mutations: R113A, K114E, R113A + K114A, K131E, R132A, or K133A) and observed loss of nuclear localization of the protein only with arginine/lysine replacements in the latter region, which was therefore proposed to work as a monopartite NLS. In addition, they found that Aire nuclear import occurs through the classical importin α/β pathway and the binding to various members of α-importin family.

In our study, we show that the p.R132S replacement in 131-KRK-133 influences the function of NLS, by displaying crystal structures of a NLS peptide containing the identical KRK basic residue cluster bound to α-importins. The central arginine (corresponding to Aire Arg132) makes a multiplicity of interactions with α-importins regardless the KRK cluster is bound to the minor or the major NLS-binding site. Serine replacement does not reproduce these interactions. Thus, we propose that the p.R132S in Aire exerts its pathogenic effect by impairing the nuclear import of the protein.

So far, about 115 *AIRE* mutations have been identified in the APS-1 syndrome (30). Most of these variants, distributed throughout coding and non-coding regions, are either nonsense or frameshift mutations that generate truncated protein sequences or single amino acid missense variants [reviewed in Ref. (41, 42)]. The diverse spectrum of variants can include single-nucleotide substitutions, insertions, and deletions with protein splicing effects. In addition, beside the conventional mutation analysis, even large genomic deletions can alter *AIRE* gene sequence [reviewed in Ref. (41, 42)]. Of note, the here reported novel *AIRE* mutation is the first NLS mutation affecting Aire shuttling to the nucleus that is associated with APS-1 syndrome.

Regarding clinical penetrance of known *AIRE* gene mutations, these are assumed to be inherited in an autosomal recessive manner. Nevertheless, p.G228W mutation of the SAND domain exceptionally follows a dominant inheritance pattern (43). Furthermore, monoallelic dominant-negative *AIRE* mutations were also reported in literature as associated with milder autoimmune phenotypes (44). Regarding the novel p.Arg132Ser mutation of the present report, the absence of autoimmune manifestations within the family and the heterozygosity of the parents suggest autosomal recessive transmission.

Epidemiological investigations have shown that *AIRE* signatures have been identified in peculiar ethnicities, i.e., in Finns [c.769C>T (R257X)], Sardinians [c.415C>T (R139X)], and Iranian Jews [c.254A>G (Y85C)] (16, 19, 20). In the light of the foregoing, expanded population studies based on *AIRE* gene screening in Pakistan would be necessary, since a very limited number of APECED patients, mostly diagnosed on clinical findings, is reported nowadays in the scientific literature (Table 2). On a general ground, definitely, genetic screening along with genetic counseling and accurate anamnesis especially in family members of consanguineous marriages would help identify heterozygote carriers of *AIRE* gene mutations in the Pakistani population (7, 41, 45). This extended approach would improve early diagnosis and avoid severe disease complications in newly affected patients. Furthermore, the relevance of the novel c.396G>C mutation would be unraveled by proving its diagnostic efficacy in association with the clinical phenotype and the detection of IFN-ω Abs in this ethnicity.

## Materials and Methods

### Molecular Studies: *AIRE* Screening

All 14 exons and intronic regions of the *AIRE* gene were sequenced according to already described protocols (Genetic Analyzer 3500 Applied Biosystems HITACHI system, Thermo Fisher Scientific, Rodano, Italy) in the DNA of the 16-year-old patient, her 46.1-year-old mother, and her 48.3-year-old father (41). Parental consent was obtained for the study.

### Structural and Conservation Analysis at the Site of the p.Arg132Ser Mutation

To display the mode of binding of the 131-KRK-133 fragment of Aire as a NLS to α-importins, we employed the crystal structures of the A89NLS peptide (VHKTVLG**KRK**YW), which carries identical cluster of positive residues, in its complex with the minor NLS-binding site of importin subunit α-1a from Oryza sativa (Protein Data Bank, PDB, 4B8P), and with the major NLS-binding site of importin subunit α-1 from mouse (PDB 4BA3). Multiple sequence alignment was made around Arg132 to highlight the conservation of this residue and of the cationic-type amino acids in the 131–133 interval characterizing the NLS of the protein. The software Muscle was used (46).

## Ethics Statement

The study was approved by the local Institutional Review Board (IRB) of Bambino Gesu Children’s Hospital, regulating the use of human samples for experimental studies (Protocol number 1385_OPBG_2017). Written informed consent was obtained from participants.

## Author Contributions

MP performed the molecular screening, the biological experiments, and helped writing the paper. EB performed the structural analysis of the mutation. RD provided medical care to the patient and presented the clinical picture of the case. FF helped in the molecular screening and analysis of the literature for the Pakistani cohort. CB collaborated in the analysis of the case and writing of the paper. AF designed the study, coordinated the work, wrote the paper, and edited the manuscript.

## Conflict of Interest Statement

The authors declare that the research was conducted in the absence of any commercial or financial relationships that could be construed as a potential conflict of interest.
