# Supplementary material for: A Novel Homozygous Mutation of the AIRE Gene in an APECED Patient From Pakistan: Case Report and Review of the Literature
Source: Front Immunol. 2018 Aug 13;9:1835. doi: 10.3389/fimmu.2018.01835 (PMC6099424; doi:10.3389/fimmu.2018.01835)
Supplement: Supplementary file 1 [file Data_Sheet_1.PDF]

Supplementary Material

A Novel Homozygous Mutation of the *AIRE* Gene in an APECED Patient from Pakistan: Case Report and Review of the Literature

Marsha Pellegrino, Emanuele Bellacchio, Rudina Dhamo, Federica Frasca, Corrado Betterle, Alessandra Fierabracci*

*** Correspondence:** Alessandra Fierabracci MD PhD: alessandra.fierabracci@opbg.net

Supplementary Figures

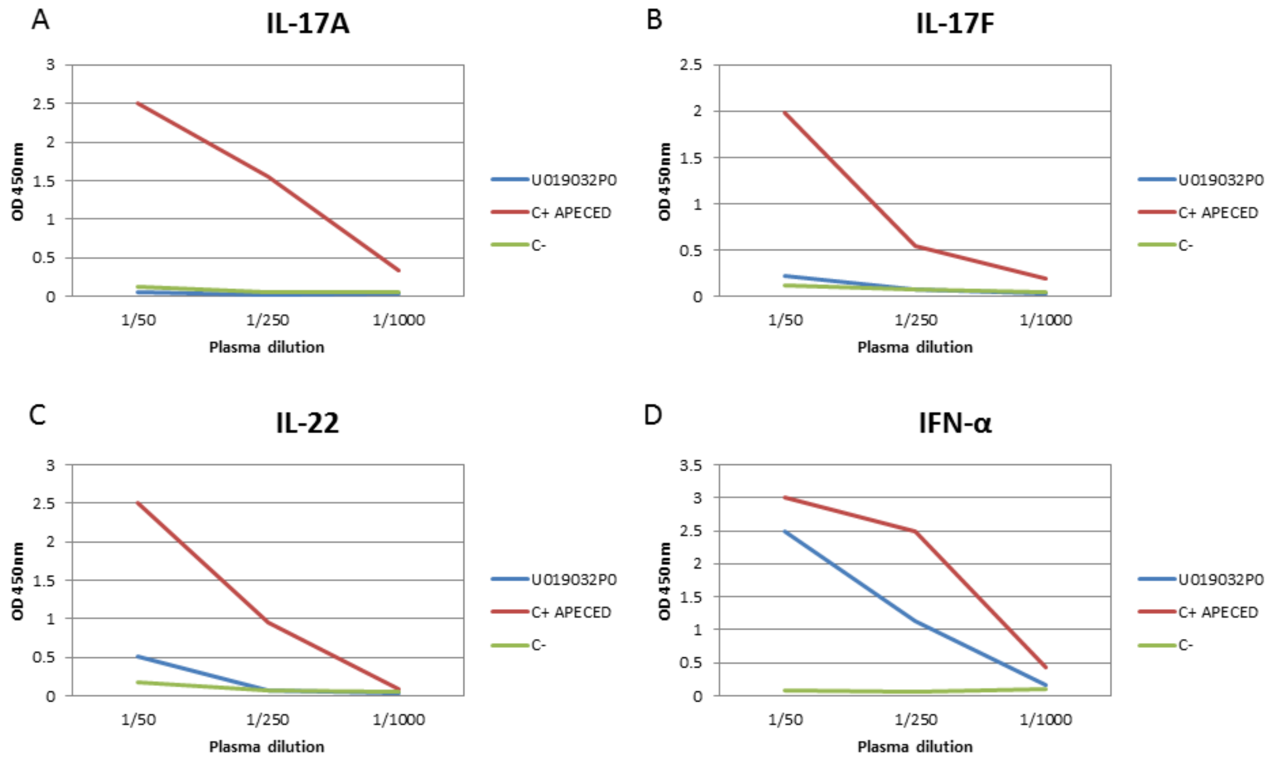

Supplementary Figure 1. Patient plasma auto-antibodies. Patient plasma (U019032P0/blue line) was tested for presence of autoantibodies against IL-17A (A), IL-17F (B) and IL-22 (C) and IFN-α (D). Positive control (C+APECED) refers to plasma of an APECED patient (red line) and negative control (C-) to a plasma of a healthy subject (green line).

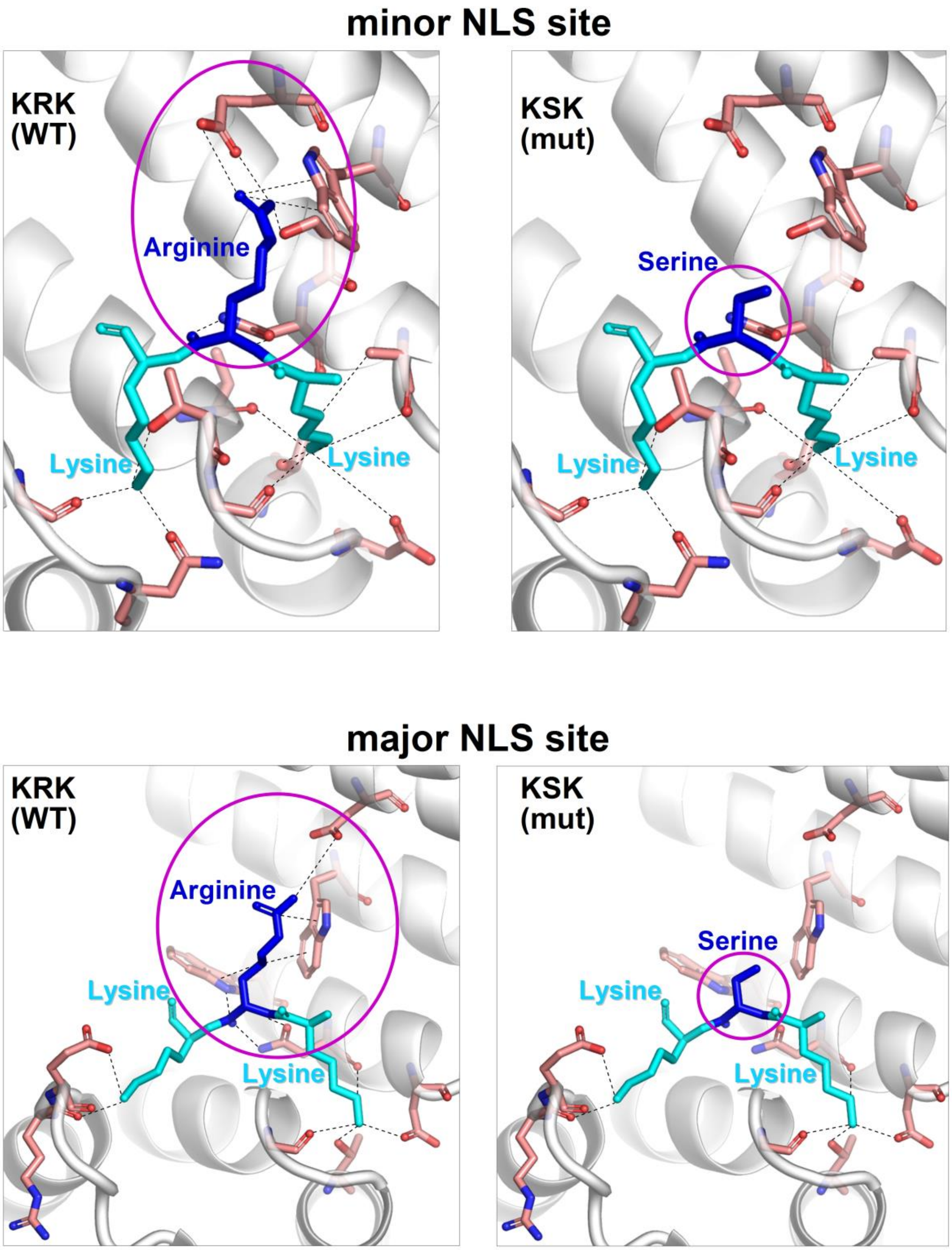

Supplementary Figure 2. Three-dimensional model of NLS interactions. Structures of the KRK portion of the A89NLS peptide (VHKTVLG**KRK**YW) complexed to α-importins at the minor NLS-binding site (top, PDB 4B8P) and at the major NLS-binding site (bottom, PDB 4BA3). The interactions between the KRK peptide and residues in the NLS-binding sites in the α-importins are indicated by dashed black lines. The arginine to serine replacement is modelled.
